# Game changing technology addresses unmet need in occlusion based shunt failures by providing a non-invasive solution to re-establish flow in occluded Ventricular Catheters

**DOI:** 10.1186/2045-8118-12-S1-O24

**Published:** 2015-09-18

**Authors:** Andrew East, Tomer Anor, Deep Singh, Morgan Brophy, Greg Eberl, PJ Anand, JR Madsen

**Affiliations:** 1 Alcyone Lifesciences, Inc., USA; 2 Boston Children's Hospital, USA

## Introduction

Ventricular catheter (VC) occlusion due to choroid plexus is the predominant cause of shunt malfunction whose prevention remains a major unsolved problem, despite best efforts of clinicians and device designers. Shunt malfunction usually demands can be life threatening, and frequently triggers urgent nocturnal surgical intervention to revise the shunt system, costing on average ~$34K per surgery. The total cost of shunt surgeries in the U.S. exceeds $2 billion per year.

Clinical experience suggests that some of the occluded VCs can be unblocked by carefully “flushing” them with saline during surgery, restoring cerebrospinal fluid (CSF) flow, so potentially a pulse of retrograde flow could dislodge choroid plexus. We therefore sought to design a retrograde flushing device, used non-invasively, hoping to avoid a need for emergency surgery.

## Methods

We therefore measured the pressure and volume ranges of the injected fluid used for flushing blocked VCs as deemed safe by expert neurosurgeons in the operating room, as approved by our IRB. Based on the surgical pressure volume impulse characteristics, an implantable device compatible with existing shunt systems was designed to simulate the surgical flushing technique. In addition to the implantable retrograde shunt flusher, a modification of the VC was designed to generate an alternative CSF flow pathway when the obstruction does not yield. Device prototypes were manufactured and extensive testing was conducted to validate design requirements.

## Results

According to measurements taken during the study, the surgeons were typically applying up to 50 PSI of pressure within the occluded VC while injecting 0.1-0.5 cc of saline with a syringe in short “pumps” attempting to unblock a VC. In some cases, the flushing maneuver was successful in increasing flow through the VC.

Through bench testing of the Alcyone prototype flushing technology, it has been demonstrated that a retrograde pulse of fluid can be generated, non-invasively by external manipulation, similar to the pressure and volume measurements taken in the OR.

## Conclusions

Alcyone shunt technology provides a non-invasive solution to re-establish flow in an occluded VCs and may extend the life of the shunt, prevent revision surgeries and avoid life-threatening emergencies caused due to blocked/non-flowing shunt complications.

